# Dorfman pooling enhances SARS-CoV-2 large-scale community testing efficiency

**DOI:** 10.1371/journal.pgph.0001793

**Published:** 2023-04-18

**Authors:** Julian Burtniak, Adam Hedley, Kerry Dust, Paul Van Caeseele, Jared Bullard, Derek R. Stein

**Affiliations:** 1 University of Manitoba, Winnipeg, Canada; 2 Cadham Provincial Laboratory, Manitoba Health, Winnipeg, Canada; University of Toronto, CANADA

## Abstract

PCR-based analysis is the gold standard for detection of SARS-CoV-2 and was used broadly throughout the pandemic. However, heightened demand for testing put strain on diagnostic resources and the adequate amount of PCR-based testing required exceeded existing testing capacity. Pooled testing strategies presented an effective method to increase testing capacity by decreasing the number of tests and resources required for laboratory PCR analysis of SARS-CoV-2. We sought to conduct an analysis of SARS-CoV-2 pooling schemes to determine the sensitivity of various sized Dorfman pooling strategies and evaluate the utility of using such pooling strategies in diagnostic laboratory settings. Overall, a trend of decreasing sensitivity with larger pool sizes was observed, with modest sensitivity losses in the largest pools tested, and high sensitivity in all other pools. Efficiency data was then calculated to determine the optimal Dorfman pool sizes based on test positivity rate. This was correlated with current presumptive test positivity to maximize the number of tests saved, thereby increasing testing capacity and resource efficiency in the community setting. Dorfman pooling methods were evaluated and found to offer a high-throughput solution to SARS-CoV-2 clinical testing that improve resource efficiency in low-resource environments.

## Introduction

The first cases of the novel Coronavirus SARS-CoV-2 were identified in Wuhan, China in December 2019 [1]. Subsequent worldwide spread and the high transmissibility of SARS-CoV-2 led the World Health Organization to declare a global pandemic on March 11, 2020. As of February 2023, over 671 million cases of SARS-CoV-2 have been detected globally [2], and thus a significant focus has been placed on reducing spread in order to decrease the morbidity associated with the disease by identifying and controlling vectors of transmission [3].

In order to facilitate contact tracing and control community transmission, widespread polymerase chain reaction (PCR) based testing has been employed to identify positive SARS-CoV-2 cases in the community. PCR-based testing remains a highly sensitive and specific lab-based test to diagnose SARS-CoV-2, and thus its use was implemented broadly early in the pandemic with high demand [3]. However, as SARS-CoV-2 transmission increased alongside case counts, the amount of testing increased as well [4]. This heightened demand put strain on many laboratories’ ability to conduct an adequate amount of diagnostic PCR-based testing as both PCR kits, PCR machines, and trained workforce were not always readily available.

In response, pooled testing strategies presented an effective method to increase testing capacity by decreasing the number of resources required for laboratory PCR analysis of SARS-CoV-2 [5]. Pooled testing was first used in 1943, as described by Robert Dorfman when faced with the problem of identifying and screening large amounts of World War II soldiers for syphilis [6]. Subsequently, pooled testing has been used successfully to screen large populations for infectious diseases. Pooled testing has been utilized in the laboratory testing of HIV, tuberculosis, hepatitis B and C, as well as the screening of blood donations to the American Red Cross [7–9]. Its utility has been demonstrated as a way to analyze large numbers of samples with greater efficiency than individual testing allows.

Dorfman pooling is the most basic pooled testing regimen, which follows a two-stage process. Dorfman testing entails combining multiple specimen samples as a single sample before PCR analysis [5]. If the pooled sample returns a negative result, then all specimens which were pooled together are resultantly negative. If a positive result is obtained for the pool, then all samples are then analyzed individually (deconvolution). Dorfman pooling allows for a higher level of test efficiency than individual sample testing, however at the expense of greater complexity of sample preparation.

Pooled testing methods for SARS-CoV-2 allow for a decrease in the number of tests used for PCR analysis. In all such pooling schemes, test efficiency is dependent on the current sample positivity rate [10]. With Dorfman pooling, the efficiency decreases with increasing test positivity due to a greater likelihood of deconvolution being necessary. Thus, the pooling size should be adapted with an ever-changing positivity rate, with a smaller pool size being more optimal alongside higher positivity rates. Pooling strategies are best used in scenarios where a large number of samples with a presumptive low positivity rate are required to be tested. SARS-CoV-2 testing presents an ideal scenario for the use of pooled testing, as test positivity remained at a level where pooling would be beneficial throughout the majority of the pandemic, and testing resources were struggling to keep up with demand using individual testing strategies.

Pooled testing methods offer a high-throughput solution to clinical testing that improves resource efficiency [11]. We sought to conduct an analysis of SARS-CoV-2 pooling schemes to determine the sensitivity of various sized Dorfman pooling strategies to evaluate the utility of using such pooling strategies in diagnostic laboratory settings.

## Methods

**Fig 1:**
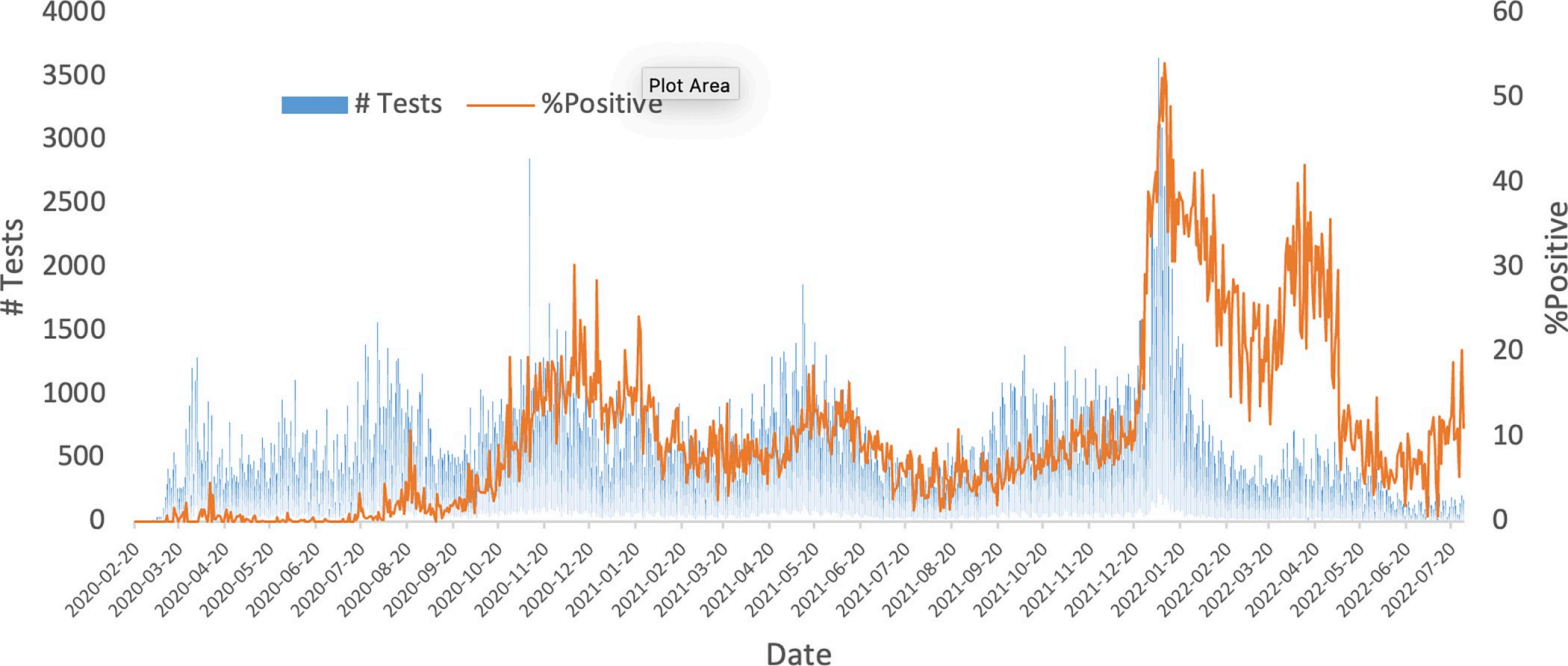
Number of tests and positivity rate at Cadham provincial laboratory. Samples were analyzed by PCR analysis, and positive tests are tabulated using results from all samples analyzed per day. Data date range February 19, 2020 to July 28 2022.

Ethics & Study design- The study was completed at Cadham Provincial Laboratory (CPL) in Winnipeg, Manitoba, Canada to analyze SARS-CoV-2 pooling schemes. CPL is the sole public health diagnostic and surveillance laboratory in Manitoba which conducted large-scale SARS-CoV-2 PCR testing. CPL routinely tested approximately 3000 clinical SARS-CoV-2 samples per day at the height of the pandemic with a peak positivity of 54% (Fig 1). CPL receives samples from populations submitted from across Manitoba. The samples eligible for inclusion in the study were positive and negative SARS-CoV-2 nasopharyngeal swabs in viral transport medium that were acquired by CPL and underwent PCR analysis from June 2021 to June 2022. This study was conducted solely under the fulfilment of ongoing SARS-CoV-2 pandemic response and is considered a quality assurance initiative exempt under the Canadian Tri-Council Policy Statement: Ethical conduct for research involving humans, TCSP2. All clinical specimens were de-identified and used solely with intent to evaluate the performance of our proposed pooling schemes.

**Fig 2:**
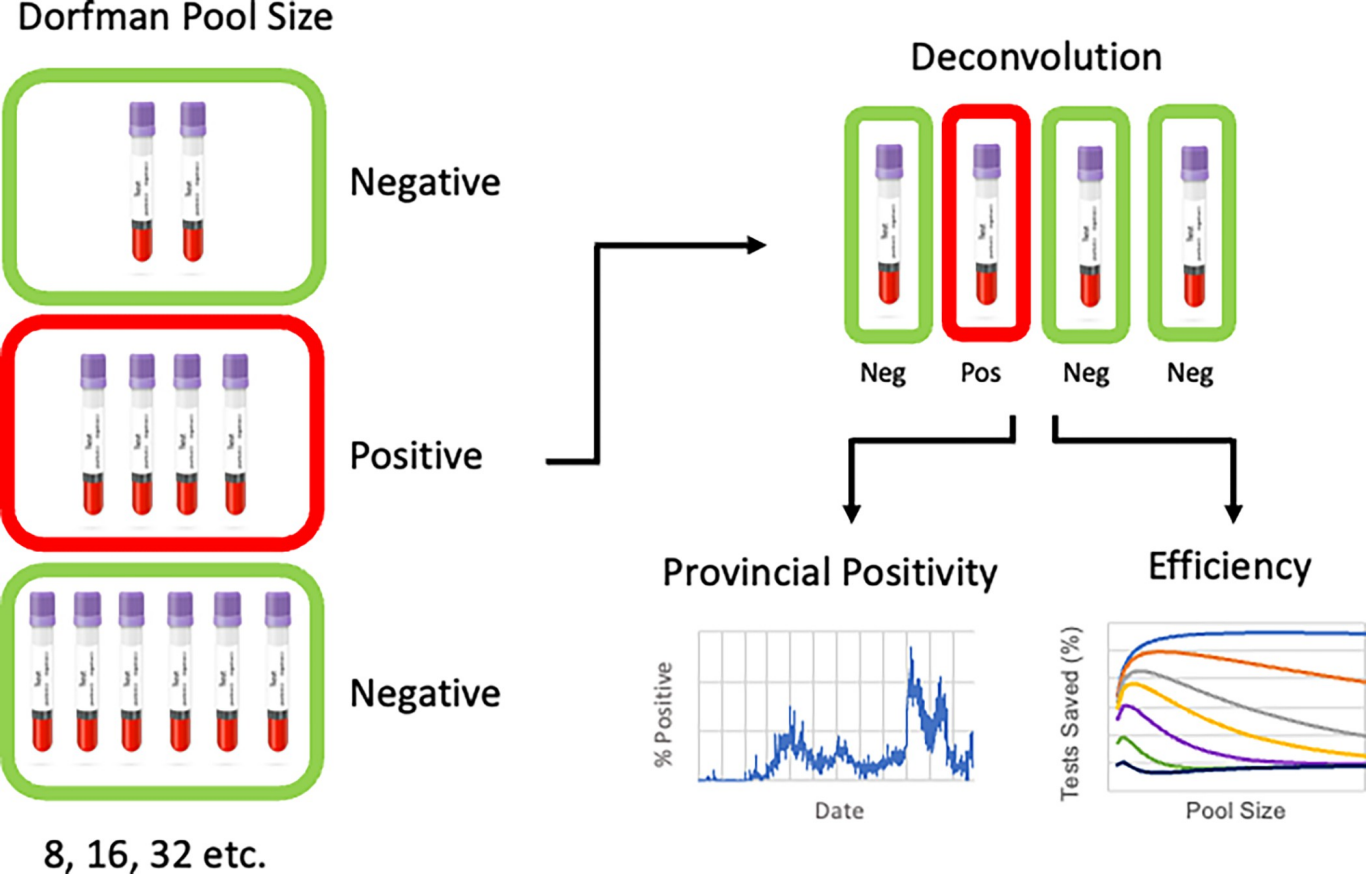
Dorfman pooling schematic. Samples are represented by tubes, and PCR tests are represented by green (negative) or red (positive) rounded rectangles. When a Dorfman pool is positive, deconvolution is required. When a positive pool is detected each sample is individually re-tested. Pool sizes of 4, for example would result in 7 total tests utilized for analysis of 12 samples. In addition, positivity rate, sensitivity, and efficiency gained need to be taken into consideration when choosing an appropriate pool size for large scale testing.

Procedures & Laboratory Analysis- All positive and negative SARS-CoV-2 nasopharyngeal specimens stored at CPL (-80°C) were eligible for inclusion in our study and retrieved at random. Samples were inactivated in a heat bath at 56°C for 30 minutes [12]. Dorfman pools were then prepared as illustrated in Fig 2 using a Hamilton Microlab Star Liquid Handling system with sizes from 2 to 32 to a final volume of 600 μl. Replicate pools were prepared for each pool size as indicated in Table 1. Each pool consisted of one positive SARS-CoV-2 sample, and the remainder of the pool was populated with negative SARS-CoV-2 samples. PCR analysis was then executed on the *cobas* 6800 system. The test system was a fully automated sample preparation (RNA extraction and purification) followed by PCR amplification and detection. PCR amplification included a unique SARS-CoV-2 target (ORF1/a non-structural region) and an additional conserved target (E-gene; envelope structural protein) for pan-Sarbecovirus detection. Upon loading patient samples on the *cobas* 6800, nucleic acid from patient samples and an internal RNA control were simultaneously extracted. Nucleic acid was released by the addition of proteinase and lysis reagent to the sample. The released nucleic acid bound to the silica surface of the added magnetic glass particles. After subsequent wash steps, purified nucleic acid was eluted from the magnetic glass particles with elution buffer at an elevated temperature. Both positive and negative external controls were processed in the same way with each *cobas* SARS-CoV-2 run. Eluted RNA was added to the PCR plate along with the *cobas* SARS-CoV-2 master mix. During amplification, each reporter dye specific for its respective target was measured at defined wavelengths, which enabled simultaneous detection and discrimination of the amplified coronavirus target and the RNA internal control. A positive test was defined as a detection of the SARS-CoV-2 target genes open reading frame 1a (ORF1/a), or envelope (E). Cycle threshold (Ct) values were assigned for each gene target, and detection of either target constituted a positive test. The Ct values for targets ORF1/a and E were recorded for the pooled samples and were compared to the Ct values of the individual samples’ test results for data analysis.

**Table 1:** Test sensitivity based on Dorfman pooling size. True positive and false negative tests are determined against gold standard of individual testing.

Dorfman Pool Size	True Positives	False Negatives	Sample Size	Sensitivity (95% CI)
2	45	2	47	95.7% (85.5–99.5)
4	46	1	47	97.9% (88.7–99.9)
6	46	1	47	97.9% (88.7–99.9)
8	46	1	47	97.9% (88.7–99.9)
16	31	6	37	83.8% (68.0–93.8)
32	31	7	38	81.6% (65.7–92.3)

Statistical Analysis- Paired Wilcoxon T-tests were used to determine the change in Ct value in pooled specimens at each pool size compared to their unpooled Ct values. Sensitivity and 95% confidence intervals for each pool size were calculated by the exact Clopper-Pearson method in comparison to individual specimen testing. Efficiency data was calculated using the calculation $Tests\;Saved={\left(1-p\right)}^{k}-\frac{1}{k}$, where *p* is positivity rate, and *k* is pool size [13]. This was used to determine optimal Dorfman pooling sizes adapted based on positivity rate. Data from this was plotted to define test efficiency curves for Dorfman pooled protocols. Optimally pooled Dorfman testing sizes based on positivity rate were calculated using the formula $n\approx Round\left\{{\left[\ln\left(\frac{1}{1-p}\right){\left(1-p\right)}^{n_{0}}\right]}^{-\frac{1}{2}}\right\}$ and n≈Round(1+1p), where *n* is pool size, *p* is infection probability, and the *Round* operator rounds the result to the nearest positive integer [14].

## Results

The study analyzed the sensitivity loss when Dorfman pooling sizes of 2, 4, 6, 8, 16, and 32 were conducted. Overall, a trend of decreasing sensitivity with larger pool sizes was observed, and pool sizes of 2, 4, 6, and 8 (n = 47/pool) maintained a high level of sensitivity. A Dorfman pool size of 2 showed a sensitivity of 95.7% (95%CI; 85.5–99.5) when compared to individual testing (Table 1). Dorfman pooling of sizes 4, 6, and 8 all showed a sensitivity of 97.9% (95%CI; 88.7–99.9) compared to individual testing. A pool size of 16 (n = 37) resulted in a sensitivity of 83.8% (95%CI; 68.0–93.8), which showed a decrease in sensitivity from the unpooled samples. Pooling of 32 (n = 38) samples showed a sensitivity of 81.6% (95%CI; 65.7–92.3), the greatest sensitivity loss of all Dorfman pooling schemes studied in comparison with individual testing. The most significant losses in sensitivity were observed at Dorman pool sizes of 16 and 32, while the 2, 4, 6, and 8 pooling sensitivities were all similarly high. All pooling sizes tested had 100% specificity compared to individual testing, with no falsely positive tests recorded across any of the pools tested.

The greatest differences in test sensitivity were seen at high Ct values (weakly positive samples), above 30. The test sensitivity of Dorfman pool sizes 2, 4, 6, 8, 16, and 32 when analyzing samples with individual Ct target E greater than 30 was 83.3, 91.7, 92.9, 92.3, 40.0, and 30.0%, respectively. With pool sizes of 16 and 32, all samples with an original Ct E greater than 34 were falsely negative. Dorfman pooling sizes of 16 and 32 showed a large loss in sensitivity in the detection of weakly positive samples in comparison with smaller pooling sizes and individual testing. However, in specimens with an original Ct value below 30 for the E gene, all pooling sizes showed zero false negatives in comparison with individual testing and 100% test sensitivity.

**Fig 3:**
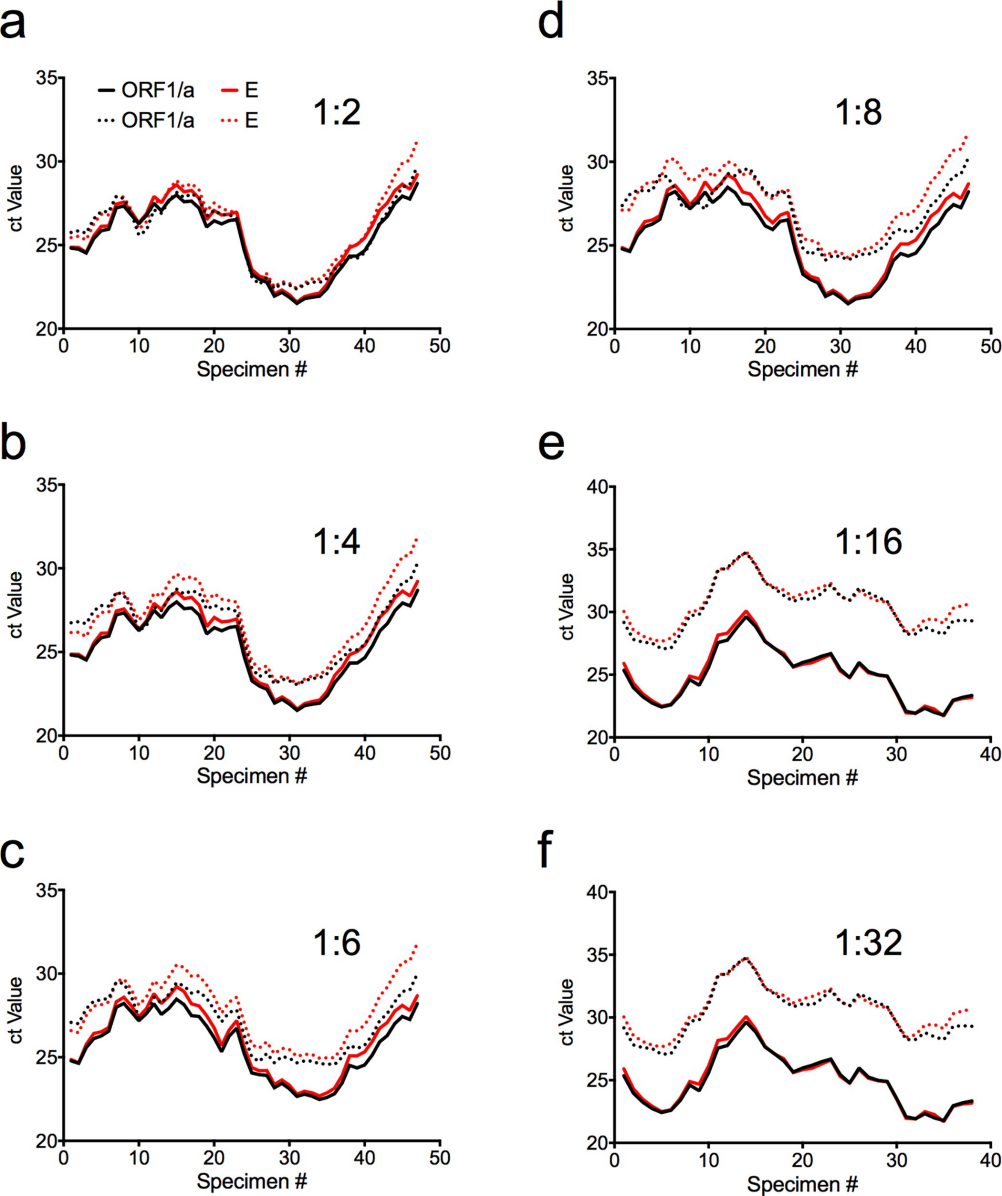
Change in Dorfman Pooled Ct values for each pool size. Individual unpooled Ct values are represented by solid lines, and pooled Ct values are graphed with dotted lines for each PCR target ORF1/a and E. Ct values are smoothed using an average of the nearest eight neighbours. A default Ct value of 40 was also instituted when pools had a result of “Target not detected” (Supplemental Material).

Paired T-testing was also conducted to demonstrate the change in Ct values between unpooled and pooled specimens. The Wilcoxon paired T-tests of Target E Ct values for 1 in 2 Dorfman pooling achieved a P-value of 0.0351. At Dorman pools of 1 in 4, 6, 8, 16, and 32, the paired T-test for Target E Ct values was <0.0001. The greatest increases in Ct values were seen at the higher pooling schemes of 16 and 32. The change in Ct E values is illustrated in Fig 3, demonstrating the greater increase in pooled Ct values at higher number pooling schemes.

**Fig 4:**
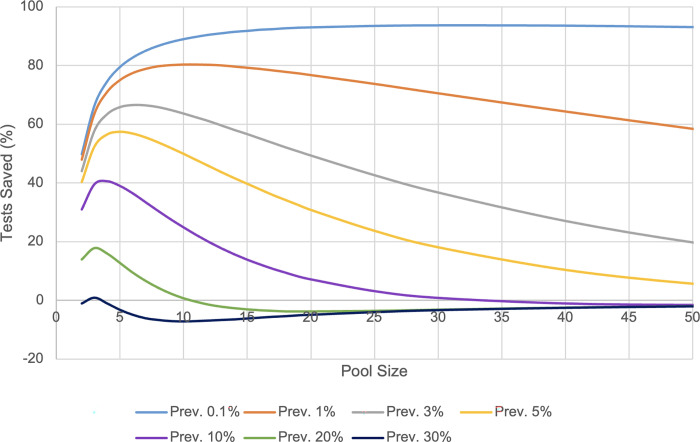
Test efficiency of Dorfman pooling. The efficiency of utilizing Dorfman pooling at varying pool sizes adapted to positivity prevalence compared to individual testing. A positive value for tests saved represents a reduction in tests used compared to individual testing, and negative values represent an increase in the number of tests used.

Efficiency data was calculated to determine the ideal Dorfman pool sizes based on test positivity rate in order to maximize test efficiency. It was calculated that the pool sizes 2, 4, 6, 8, 16, and 32 are most efficient at positivity rates of 45, 10, 4, 2, 0.4, and 0.1%, respectively. Efficiency curves of pool sizes at positivity rates of 0.1, 1, 3, 5, 10, 20, and 30% are illustrated in Fig 4. This showed the trend of pooling schemes across all pooling sizes having highest efficiency at lower prevalence, and decreasing when test positivity increases.

## Discussion

The results of this study showed sensitivity losses across all sizes of pooling schemes, however, the 2, 4, 6, and 8 sized schemes were highly sensitive, while the most significant sensitivity losses were associated with the 16 and 32 sized Dorfman pooling. This data can be used to clinically guide which pooling scheme is to be utilized, gauged by acceptable sensitivity and false negative rate. A trade-off of test sensitivity for higher resource efficiency can be considered in resource-limited settings in order to facilitate a higher amount of test processing. In settings where a lower test sensitivity is acceptable due to constraints on testing resources and testing capacity, Dorfman pooling schemes in excess of 32 samples could be considered to facilitate a higher quantity of testing, and therefore higher levels of case detection overall. In settings where high sensitivity is required, then smaller pooling schemes of 2, 4, 6, and 8 can be used effectively, with resource efficiency being dependent on current test positivity.

The false negative samples at each pooling size tested were associated with samples that were weakly positive (yielded a high Ct value) when individually tested; all Ct values analyzed as falsely negative had a target E Ct of 33 or greater. The random sampling approach we took in this study mimicked real life sampling from a large community population, resulting in an additional false negative sample for the pool size of 2 compared to pool sizes 4, 6, and 8. This resulted in a slightly lower sensitivity for pool size 2 (95.7%) compared to 4, 6, and 8, however given the confidence intervals overlap it is expected that all 4 pooling schemes would have similar sensitivity performance in practice. The Ct values of the falsely negative samples are of importance, as infectivity is inversely related to Ct value. It has been demonstrated that SARS-CoV-2 infectivity is significantly reduced when PCR target E values are greater than 24 [15]. This can guide the implementation of large-scale pooling schemes within acceptable levels of sensitivity loss in detecting weak positive samples with decreased infectivity. These results are supported by previous studies on Dorfman pooling by *Bateman* and *Alcoba-Florez et al* [16,17]. Similar levels of sensitivity loss with increasing pool size were observed, with high Ct values being the most likely to be analyzed as falsely negative when pooled. The most significant increases in Ct value were observed at pooling sizes of 16 and 32. This is in agreement with the study sensitivity results, as the greater Ct value increases at larger pooling sizes causing a greater likelihood of pushing the high Ct (weakly positive) samples over the PCR cycle threshold cut-off, yielding a lower sensitivity and more falsely negative samples. Importantly, all pooling schemes tested showed a sensitivity of 100% when original target E Ct values were below 33. This demonstrates a high reliability to detect cases which are strongly positive for SARS-CoV-2, and have a higher level of infectivity than those with higher Ct values which are more likely to be falsely negative [15]. It is essential to detect these cases quickly and efficiently, and pooled testing provides a method to do so that can be implemented effectively in a large-scale clinical laboratory.

Pooling efficiency varies by positivity rate, and thus it is valuable to adapt the pooling size utilized based on the expected percentage of positive tests. It was revealed that smaller size pooling schemes are most efficient at high levels of test positivity, and large pooling schemes can be employed most efficiently at low levels of test positivity (**Fig 4**). This is attributed to the higher likelihood of deconvolution being necessary with increasing test positivity rate. This information can be used to guide decision making when employing large-scale community testing. Tailoring pool size to the presumptive positivity rate helps yield optimal testing efficiency in resource-limited settings. **Fig 2** demonstrates the dramatically changing positivity rate of samples analyzed by CPL since the beginning of the pandemic. Closely monitoring such trends will allow the greatest resource efficiency in the implementation of Dorfman pooling by correlation with optimal pool size (**Fig 4).**

Other pooling strategies in addition to Dorfman pooling have been used in clinical testing. Single-stage pooling strategies can be utilized, in which samples are pooled and analyzed without the need for deconvolution. Tapestry pooling is a type of single-stage pooling strategy that operates by analyzing each sample in three different pools [18]. This is advantageous as the deconvolution step is then avoided by algorithmically decoding the array of positive results, and no second-stage testing is required. As with Dorfman pooling, single-stage pooling strategies such as Tapestry pooling allow for a high level of test efficiency, however at the expense of greater complexity of sample preparation.

This study has potential limitations. The first being that nasopharyngeal samples were stored at -80°C prior to being thawed and analyzed. It is possible that sample degradation occurred and influenced results. The second limitation concerns the evolving nature of SARS-CoV-2. The variants circulating in the population at the time of the study included predominantly the Delta and Omicron waves, and thus future variants were not tested. However, we do not anticipate changes in clinical performance based on the variants circulating as the cobas 6800 targeted conserved regions of E and ORF1/a, rather than primers which target the SARS-CoV-2 spike protein, which would be more susceptible to newer variants’ spike mutations.

Dorfman pooling has important clinical value in large-scale PCR testing for SARS-CoV-2 in low-resource settings. Dorfman pooling can be utilized optimally when tailored to test positivity and guided by an acceptable sensitivity based on pool size; this study can guide the implementation of Dorfman pooled testing in the clinical laboratory for greater resource efficiency in widespread testing.

## Supporting information

S1 Data (XLSX)
